# Analogous Mechanisms of Resistance to Benzothiazinones and Dinitrobenzamides in *Mycobacterium smegmatis*

**DOI:** 10.1371/journal.pone.0026675

**Published:** 2011-11-01

**Authors:** Ana Luisa de Jesus Lopes Ribeiro, Giulia Degiacomi, Fanny Ewann, Silvia Buroni, Maria Loreto Incandela, Laurent R. Chiarelli, Giorgia Mori, Jaeseung Kim, Monica Contreras-Dominguez, Young-Sam Park, Sung-Jun Han, Priscille Brodin, Giovanna Valentini, Menico Rizzi, Giovanna Riccardi, Maria Rosalia Pasca

**Affiliations:** 1 Dipartimento di Genetica e Microbiologia, Università degli Studi di Pavia, Pavia, Italy; 2 Dipartimento di Scienze Chimiche, Alimentari, Farmaceutiche e Farmacologiche, Università del Piemonte Orientale “A. Avogadro”, Novara, Italy; 3 Biology of Intracellular Pathogens Inserm Avenir, Institut Pasteur Korea, Bundang-gu, Seongnam-si, Gyeonggi-do, Korea; 4 Dipartimento di Biochimica “A. Castellani”, Università degli Studi di Pavia, Pavia, Italy; National Institute for Infectious Diseases (L. Spallanzani), Italy

## Abstract

Tuberculosis is still a leading cause of death worldwide. The selection and spread of *Mycobacterium tuberculosis* multidrug-resistant (MDR-TB) and extensively drug-resistant strains (XDR-TB) is a severe public health problem. Recently, two different classes of chemical series, the benzothiazinones (BTZ) and the dinitrobenzamide (DNB) derivatives have been found to be highly active against *M. tuberculosis*, including XDR-TB strains. The target of BTZs is DprE1 protein which works in concert with DprE2 to form the heteromeric decaprenylphosphoryl-β-D-ribose 2′-epimerase, involved in Decaprenyl-Phospho-Arabinose (DPA) biosynthesis. Interestingly, it has been shown that the DNBs block the same pathway thus suggesting that both drugs could share the same target. Moreover, in *Mycobacterium smegmatis* the overexpression of the NfnB nitroreductase led to the inactivation of the BTZs by reduction of a critical *nitro*-group to an *amino*-group. In this work several spontaneous *M. smegmatis* mutants resistant to DNBs were isolated. Sixteen mutants, showing high levels of DNB resistance, exhibited a mutation in the Cys394 of DprE1. Using fluorescence titration and mass spectrometry it has been possible to monitor the binding between DprE1 and DNBs, achieving direct evidence that MSMEG_6382 is the cellular target of DNBs in mycobacteria. Additionally, *M. smegmatis* mutants having low levels of resistance to DNBs harbor various mutations in *MSMEG_6503* gene encoding the transcriptional repressor of the nitroreductase NfnB. By LC/MS^2^ analysis it has been demonstrated that NfnB is responsible for DNB inactivation. Taken together, our data demonstrate that both DNB and BTZ drugs share common resistance mechanisms in *M. smegmatis*.

## Introduction

Tuberculosis, an infectious disease caused by *Mycobacterium tuberculosis*, is still a leading cause of death in developing countries and a resurgent disease in developed countries [1]. The selection and increasing spread of *M. tuberculosis* multidrug-resistant (MDR-TB), extensively drug-resistant (XDR-TB) and, more recently, totally drug-resistant (TDR) or super-XDR strains is a serious threat to public health, in particular to immunocompromised patients [2]–[5].

For these reasons, there is an urgent need of new drugs for tuberculosis treatment with novel mechanisms of action and a necessity to identify new drug targets [4]. A major international effort has created a pipeline of new drug candidates at various stages of preclinical and early clinical evaluations [6]. Moxifloxacin and gatifloxacin are currently in phase three of clinical trials, while PA-824 and TMC207 are in phase two and SQ109, AZD5847, and linezolid in phase one [7].

Recently, new and effective antitubercular drugs, belonging to the class of benzothiazinones (BTZs), have been found to be highly active against *M. tuberculosis* resistant and sensitive strains, including MDR and XDR strains. It has been demonstrated that BTZs rapidly kill *M. tuberculosis in vitro*, *ex vivo*, and *in vivo* [8].

The cellular target of BTZs in *M. tuberculosis* is the enzyme DprE1 which is part of the decaprenylphosphoryl-β-D-ribose 2′-epimerase together with DprE2. DprE1 is encoded by the *dprE1* gene, also named *Rv3790* [8]. DprE1 and DprE2 are involved in the conversion of decaprenylphosphoryl-*D-*ribose (DPR) to decaprenylphosphoryl-*D-*arabinose (DPA), which is the only *araf* donor for arabinogalactan and lipoarabinomannan synthesis in the mycobacterial cell wall [9]–[11].

Moreover recently Crellin and collaborators (2011) demonstrated the essentiality of *M. smegmatis dprE1* (or *MSMEG_6382*) [12]. This result, combined with the conservation of *dprE1* in all sequenced mycobacterial genomes, suggests that decaprenylphosphoryl arabinose synthesis is essential in all mycobacteria [12].

It was shown that BTZs specifically react with the cysteine residue at position 387 in the DprE1 active site leading to the formation of a covalent complex between BTZs and DprE1 [13].

Additionally, in all the *M. tuberculosis* BTZ resistant mutants found to date, Cys387 of DprE1 was replaced by serine or glycine unravelling the key role played by Cys387 in BTZ bactericidal activity. The cysteine is a highly conserved amino acid residue also in orthologous enzymes from various Actinobacteria and its replacement by alanine and serine in *Mycobacterium avium* and *Mycobacterium aurum,* respectively, is the cause of their natural resistance to BTZ [8].

The benzothiazinone BTZ043, which contains a *nitro*-group in its molecule, is a very effective agent against mycobacteria; on the other hand, the corresponding *amino*-derivative, found in the blood and urine of treated animals, is significantly less active in inhibiting both *M. tuberculosis* and *Mycobacterium smegmatis* growths [8]. The reduction of the critical *nitro* group into an *amino* group has been reported in *M. smegmatis* mutants resistant to BTZ, overexpressing the nitroreductase NfnB [14]. The crystal structure of NfnB in complex with its essential cofactor flavin mononucleotide shows that an amino acid stretch which is likely to be essential for the interaction with BTZ is also contained into DprE1 enzyme. No *nfnB* ortholog could be found in *M. tuberculosis* genome and until now the pathogen seems to lack nitroreductases able to carry out BTZ inactivation [14].

Another new class of potent antitubercular drugs is represented by the dinitrobenzamide derivatives (DNBs),whose activity was identified through an *ex vivo* screening of chemicals which interfere with *M. tuberculosis* replication within macrophages [15], [16]. Incubation of *M. tuberculosis* with DNBs resulted in the inhibition of the synthesis of decaprenylphosphoryl-*D-*arabinose [15], suggesting that DprE1 could be the cellular target not only of BTZ, but also of DNB drugs.

The most active compounds among DNBs exhibit a *nitro* group at positions 3 and 5 in the benzene moiety. The two major compounds from this series, [N-(2-(4-methoxyphenoxy) ethyl)-3,5-dinitrobenzamide] and [N-(2-(benzyloxy) ethyl)-3,5-dinitrobenzamide], named DNB1 and DNB2 respectively, are also highly active against *M. tuberculosis* MDR and XDR strains [15].

In order to understand if DNBs can be suitable candidates for the antitubercular therapy, one of the main points is to know the mechanism by which they fulfill their activity at the cellular level. To this purpose, through microbiological, genetic and biochemical approaches, we investigated the molecular bases of the resistance to DNBs in *M. smegmatis*. According to two different levels of resistance, two different mechanisms were found which paralleled those exhibited by BTZ compounds.

## Materials and Methods

### Bacterial strains and growth conditions

All cloning steps were performed in *Escherichia coli* XL1-Blue, following standard methods [17]. The oligonucleotides used for all PCR amplifications are shown in Table 1.

**Table 1:** Oligonucleotides used in this work.

Oligonucleotides	Sequence (5′-3′)	Purpose
**OXRE1**	GATGTCGCTCAGTTGCACC	Sequencing of *MSMEG_6382* gene
**OXRE2**	ACCTGTTCTGGGCGACCGT	
**OXRE3**	GTGTAGTTCGCCTCGCTGC	
**OXRE4**	AGGCTACTGCTTTTCTGGA	
**3791smF**	CAAGGTGATGGACGCCGAC	Sequencing of *MSMEG_6385* gene
**3791smR**	CGCGTGCAGCTGGTTGGAC	
**AL52tetFOR**	CCGCGCCGCTGACCACCTCG	Sequencing of *MSMEG_6503* gene
**AL52tetREV**	CATCGGCTACGTCTTCGCGCAC	
**NfnBfor**	TATCTAGACGGGCAGGCACATCG	Sequencing of *nfnB* gene
**NfnBrev**	CGGGATCCTAACGGGTCAGCGG	
**nfnBinF**	GGCCAGGTCGACAGTGGGC	Amplification of *MSMEG_6503-nfnB* intergenic region
**nfnBinR**	GGCGGCCGCGGCGACGAT	
**mysAF**	CGTCGCCGATGGTCTG	Real-Time PCR
**mysAR**	CCACGCCCGAAGAGC	
**nfnBF**	GCGGTTCTACGGTGCCCC	
**nfnBR**	CGGTCATCGCGAGCATCAG	

For expression studies, the strain utilized was *E. coli* BL21(DE3), which was grown either in Luria-Bertani (LB) broth or on LB agar.

*M. smegmatis* strains were grown aerobically at 37°C with orbital shaking at 200 rpm either in Middlebrook 7H9 medium (Becton Dickinson) or on Middlebrok 7H11 agar (Becton Dickinson), both supplemented with 10% OADC Middlebrook Enrichment.

For *E. coli* cultures, when necessary, antibiotics were added at the following concentrations: ampicillin,100 µg/ml; chloramphenicol, 34 µg/ml; and kanamycin, 50 µg/ml; both DNB and BTZ were dissolved in dimethyl sulfoxide (DMSO).

### *M. smegmatis* resistant mutants

The isolation of *M. smegmatis* mutants was performed by plating ∼10^10^ cells from an exponential growth phase wild-type culture onto 7H11 medium containing different concentrations of *N*-(2-(3-chlorobenzyloxy)ethyl)-3,5-dinitrobenzamide (DNB3), ranging from 8 to 300-fold the Minimal Inhibitory Concentration (MIC) for the wild-type strain. DNB3 was chosen over DNB1 and DNB2 because of its increased solubility in agar medium. Plates were incubated at 37°C for 10 days. The phenotype of the resistant colonies hereafter referred as DNB^R-High^ and DNB^R-Low^ was confirmed by determining the MIC of DNB3; the experiment was repeated three times. AL49, AL55 and GM22 mutants, that had been selected for their resistance to BTZs, had previously been reported [14].

### MIC determinations

A single colony of each mycobacterial strain was inoculated in complete Middlebrook 7H9. Cell cultures were grown at 37°C until exponential growth phase (∼10^8^ CFU/ml) was reached. After dilution to the final concentration of about 10^6^ CFU/ml, 1 µl of cell cultures was streaked onto plates containing two-fold serial dilutions of appropriate compounds. MIC values were assigned as the lowest drug concentrations inhibiting bacterial growth. All experiments were repeated three times.

### DNA sequencing of *MSMEG_6382*, *MSMEG_6385*, *MSMEG_6503*, and *nfnB* genes from *M. smegmatis* isolated mutants

*MSMEG_6382*, *MSMEG_6385*, *MSMEG_6503*, and *nfnB* genes were amplified by PCR, using primers reported in Table 1 and PFU *Taq* DNA polymerase (Promega), according to the manufacturer's instructions. The bacterial lysates from *M. smegmatis* mutants were used as templates. The PCR products were purified using the Wizard SV Gel and PCR clean-up system (Promega) and directly sequenced.

### Real Time PCR analysis

RNA was isolated from *M. smegmatis* wild-type and mutant strains by using RNeasy Mini Kit (Qiagen). After DNase treatment (Ambion), all samples were tested by conventional PCR to eliminate DNA contamination. 1 µg of total RNA was reverse-transcribed with Quantitect reverse transcription kit (Qiagen), according to the manufacturer's instructions. Samples corresponding to 50 ng of RNA were used in each PCR reaction in a final volume of 15 µl. Quantitative PCR were performed using QuantiTect SYBR Green PCR Master Mix (Qiagen) on a Rotor Gene 6000 (Corbett Life Science). The primer pairs used were as follows: mysAF and mysAR for *mysA*; nfnBF and nfnBR for *nfnB* (Table 1). *mysA* (*MSMEG_2758*), coding for σ^A^ factor, was used as an internal invariant control for the normalization of *nfnB* expression levels. Expression data were calculated with the -2^ΔΔCt^ method (ΔC_t_  =  C_t sample_ – C_t control_) and were reported as -fold change in gene expression of the sample (mutant strain) normalized to the invariant gene (*mysA*) relative to the control wild-type strain.

### Cloning, expression and purification of recombinant DprE1 from *M. smegmatis* (MSMEG_6382)

The *MSMEG_6382* gene, encoding DprE1 in *M. smegmatis*, was cloned into *Hind*III/pET-32b vector (Novagen), to obtain the corresponding protein fused to six histidine residues and thioredoxin protein at its N-terminus. Briefly, *E. coli* BL21(DE3)pLysS cells transformed with *MSMEG_6382*/pET-32b construct were grown onto LB agar containing ampicillin (100 µg/ml). Roughly 100 colonies were inoculated in ZYP-5052 auto-inducing medium supplemented with ampicillin at 37°C for three hours and subsequently at 17°C O/N. Bacterial cells were then collected by centrifugation, resuspended in lysis buffer (50 mM NaH_2_PO_4_, 300 mM NaCl, and 10 mM imidazole, pH 8.0) supplemented with protease inhibitor cocktail (Sigma), sonicated at 800 W for 6 minutes, cleared by ultracentrifugation, and the supernatant was applied to a HisTrap HP column (GE-Healthcare) equilibrated in the same buffer. Proteins were eluted with scalar concentrations (20 to 500 mM) of imidazole, and fractions containing the DprE1 protein, as detected by 10% SDS-PAGE, were collected, dialyzed O/N at 4°C against 50 mM Tris, 150 mM NaCl, 1 mM EDTA and 1 mM DTT, pH 7.0, and incubated for 12 hours at 4°C in the presence of 10 units of PreScission Protease (GE Healthcare). The digested protein was finally concentrated and applied to a HiLoad 16/60 Superdex-200 column (GE-Healthcare) equilibrated in 50 mM potassium phosphate pH 8.0, 100 mM KCl. The enzyme was eluted by the same buffer and fractions containing DprE1 were pooled.

### Fluorescence titrations

Steady state fluorescence titration of DprE1 was performed using 2–6 µM of protein, in 20 mM potassium phosphate buffer, pH 7.5, in the presence of different concentrations of DNB1 or DNB3 (from 0 to 256 µM). Measurements were performed at 25°C with a Jasco FP-6500 spectrofluorimeter (Jasco Europe, Cremalla, Italy). The excitation wavelength was 450 nm (3 nm slit width) and the emission was recorded between 470 and 600 nm (10 nm slit width). Spectra were corrected for the blanks, performed in the same conditions, but in the absence of protein. All experiments were performed at least in duplicate. The dissociation constants (Kd) were calculated using the following equation (1), with the Sigma Plot 7.0 software (SPSS Inc):
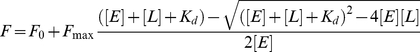
(1)where F is the observed fluorescence intensity, [E] is the enzyme concentration, [L] is the ligand concentration, F_0_ and F_max_ are the fluorescence intensity at [L] = 0 and the total change in fluorescence intensity, respectively, and Kd is the fitted dissociation constant.

### LC/MS^2^ analysis of DNB metabolized by NfnB

5 µg of NfnB protein expressed and purified as previously described [14] were incubated in 50 mM MOPS, pH = 7.9, 5 mM 2-mercaptoethanol, 10 mM MgCl_2_ buffer, and 80 µM NADPH. After addition of DNB1 at a final concentration of 7 µM, the reaction was carried out for 30 min at 37°C (each experiment was also performed in the absence of either NfnB or NADPH).

The presence of a *hydroxyl amine* and of *nitro* reduced forms of the drugs were analysed by LC/MS/MS. Each experiment was performed in triplicate. The data shown are the mean value obtained.

Chromatographic separation was performed using a Waters Acquity Ultra Performance LC system (Waters, milford, MA, USA) equipped with an Acquity UPLC™ BEH C_18_ column (100 mm × 2.1 mm, i.d., 1.7 µm particle size, Waters, Milford, MA, USA). A linear gradient at a flow rate of 0.3 ml/min was used for the elution. Mobile phases were: A; 0.1% formic acid and B; acetonitrile containing 0.1% formic acid. Gradient condition was: 0–4 min, 20-90% B in A (linear); 4–5 min, 90% B in A (isocratic); 5–6 min, 90-20% B in A (linear); 6–7 min, 20% B in A (isocratic). The separated compounds were detected by a Waters Micromass Quattro Premier XE tandem quadrupole mass spectrometer (Waters, Manchester, UK). The instrument was operated using [M+H]^+1^ and [M-H]^-1^ ionization modes. The following method parameters were used for the DNB1, DNB1-NH_2_, and DNB1-NHOH analysis; ion source temperature, 100°C; desolvation temperature, 300°C; desolvation gas flow, 750 L/h (nitrogen); Cone gas flow, 100 L/h (nitrogen); capillary voltage, 5 kV. 2 0V cone voltage and 20eV collision energy were used for [M+H]^+1^ mode and 30V cone voltage and 20eV collision energy were used for [M-H]^-1^ mode, respectively and argon was used as collision gas at a pressure of 3.0×10^−3^ mbar.

## Results and Discussion

### Isolation and characterization of *M. smegmatis* resistant mutants

To better understand the mechanism of resistance to DNBs, several spontaneous *M. smegmatis* mutants resistant to DNB3 were isolated. DNB3 was chosen because of its higher solubility in solid medium in respect to the other dinitrobenzamide derivatives.

The spontaneous mutants exhibited two different resistance levels to DNB3. The first series of *M. smegmatis* DNB resistant mutants were isolated onto 7H11 containing drug concentrations ranging from 150 to 300-fold the MIC for the wild-type strain (0.25 µg/ml) (Table 2) and the second series with concentrations ranging from 8 to 24-fold the MIC (Table 3).

**Table 2:** Resistance profile and mutations in *M. smegmatis* mutants showing high levels of resistance to DNB.

*M. smegmatis* strain	Mutation in *MSMEG_6382*	DNB3MIC (µg/ml)	BTZ-043MIC (µg/ml)
**mc^2^155**	Cys394	0,25	0,004
**DNB^R-High^1**	Cys394 → Gly	>200	4
**DNB^R-High^1**	Cys394 → Gly	>200	4
**DNB^R-High^2**	Cys394 → Gly	>200	4
**DNB^R-High^3**	Cys394 → Gly	>200	4
**DNB^R-High^4**	Cys394 → Ser	>200	>16
**DNB^R-High^5**	Cys394 → Gly	>200	4
**DNB^R-High^6**	Cys394 → Gly	>200	4
**DNB^R-High^7**	Cys394 → Gly	>200	4
**DNB^R-High^8**	Cys394 → Gly	>200	4
**DNB^R-High^9**	Cys394 → Gly	>200	4
**DNB^R-High^10**	Cys394 → Gly	>200	4
**DNB^R-High^11**	Cys394 → Gly	>200	4
**DNB^R-High^12**	Cys394 → Gly	>200	4
**DNB^R-High^13**	Cys394 → Gly	>200	4
**DNB^R-High^14**	Cys394 → Gly	>200	4
**DNB^R-High^15**	Cys394 → Gly	>200	4

**Table 3:** Resistance profile and mutations in *M. smegmatis* mutants showing lower levels of resistance to DNB.

*M. smegmatis* strain	Mutation in MSMEG_6503	DNB3 MIC (µg/ml)	BTZ-043 MIC (µg/ml)
**mc^2^155**	-	0.25	0.004
**GM22^(11)^**	Leu 137 → Pro	64	0.128
**AL49^(11)^**	ΔAla 15, 16, 17	64	0.128
**AL55^(11)^**	Mutation in the repressor binding site	64	0.256
**DNB^R-Low^1**	ΔAla 18 – Ala 108	32	0.064
**DNB^R-Low^2**	Thr51Aspfs*46	16	0.064
**DNB^R-Low^3**	Gln 61 → Stop	32	0.064

To confirm the phenotype of these resistant strains, the MIC of DNB3 was evaluated as described in Materials and Methods. Moreover, the possible cross-resistance between DNBs and BTZ043 was checked for all *M. smegmatis* mutants isolated in this work and also for three *M. smegmatis* BTZ043 resistant mutants isolated in the previous work, GM22, AL49 and AL55 [14]. All mutants showed cross-resistance to BTZs and DNBs (Tables 2 and 3).

The confirmed MICs for the first series of mutants showed a high level of resistance to DNB (>800 X MIC) and to BTZ (≥1000 X MIC) (Table 2); the second series of mutants presented a lower level of resistance to DNB (64-128 X MIC) and to BTZ (16-64 X MIC) (Table 3). The *M. smegmatis* mutants GM22, AL49 and AL55, characterized by a low level of resistance to BTZ043 [14], were also resistant to DNB (256 X MIC) (Table 3).

DNBs have been reported to inhibit DPA biosynthesis [15]. Therefore, all the DNB resistant mutants were screened for the presence of mutations in *MSMEG_6382* gene (ortholog of *M. tuberculosis Rv3790*), which codes for DprE1, a component of the heteromeric decaprenylphosphoryl-β-D-ribose 2′-epimerase. *MSMEG_6382* gene was amplified by PCR and sequenced from genomic DNA of all *M. smegmatis* mutants.

All the *M. smegmatis* mutants with a higher level of resistance to DNB (DNB^R-High^) (and also to BTZ) had a point mutation at codon 394 of *MSMEG_6382* gene, as previously observed for *M. smegmatis* mutants resistant to BTZ [8]. This position (Cys394) corresponds to Cys387 position in *M. tuberculosis Rv3790* gene. All mutants turned out to have the substitution Cys394Gly in DprE1, but DNB^R-High^4 showed a serine instead of cysteine at the same position and higher BTZ resistance than the other ones (>4000 X MIC) (Table 2). These data support the idea that Cys394 is involved in the binding of DNB, as previously suggested for BTZ [8].

Taken together, these results give clear evidence that MSMEG_6382 is a target of DNB drugs and target modification confers a high level of resistance to these compounds.

In opposition to the aforementioned strains, mutants less resistant to DNB and to BTZ (DNB^R-Low^1-3,Table 3) did not show mutations in *MSMEG_6382* gene, thus suggesting that their resistance should be accomplished by a different molecular mechanism.

*MSMEG_6385* gene (ortholog of *M. tuberculosis Rv3791* or *dprE2*) encodes the second component (DprE2) of the heteromeric decaprenylphosphoryl-β-D-ribose 2′-epimerase [10]. Given that a possible mutation in this gene could affect the binding of the drugs to DprE1, thus causing resistance, *MSMEG_6385* gene of mutants DNB^R-Low^1-3 (Table 3) was sequenced. No mutations were found, excluding the involvement of DprE2 in the mechanism of DNB resistance.

Recently, a new mechanism of BTZ resistance was described [14] for a few *M. smegmatis* mutants (GM22, AL49 and AL55; Table 3) where the nitroreductase NfnB overexpression is responsible for BTZ inactivation because of a mutation in *MSMEG_6503* gene coding for *nfnB* transcriptional repressor [14]. Thus, in order to identify the gene(s) responsible for the resistance phenotype in these DNB resistant mutants, *nfnB* and *MSMEG_6503* genes were amplified and sequenced. No one mutant had a mutation in *nfnB* gene, but all the mutants DNB^R-Low^ showed a mutation, although different from each other, in *MSMEG_6503* regulatory gene (Table 3).

DNB^R-Low^1 mutant presented a deletion of 273 nucleotides (Δ38-310); mutant DNB^R-Low^2 showed an insertion of a C at position 147; and mutant DNB^R-Low^3 had a substitution of C into T at position 181 leading to a replacement of codon for Gln61 by a Stop codon. In all cases, mutations resulted in truncated forms of the repressor protein. Therefore, the overexpression of the nitroreductase NfnB caused by the altered repressor of the *nfnB* gene could account for the resistance to this new class of antitubercular drugs in DNB^R-Low^ mutants.

### DNB - DprE1 interaction

**Figure 1:**
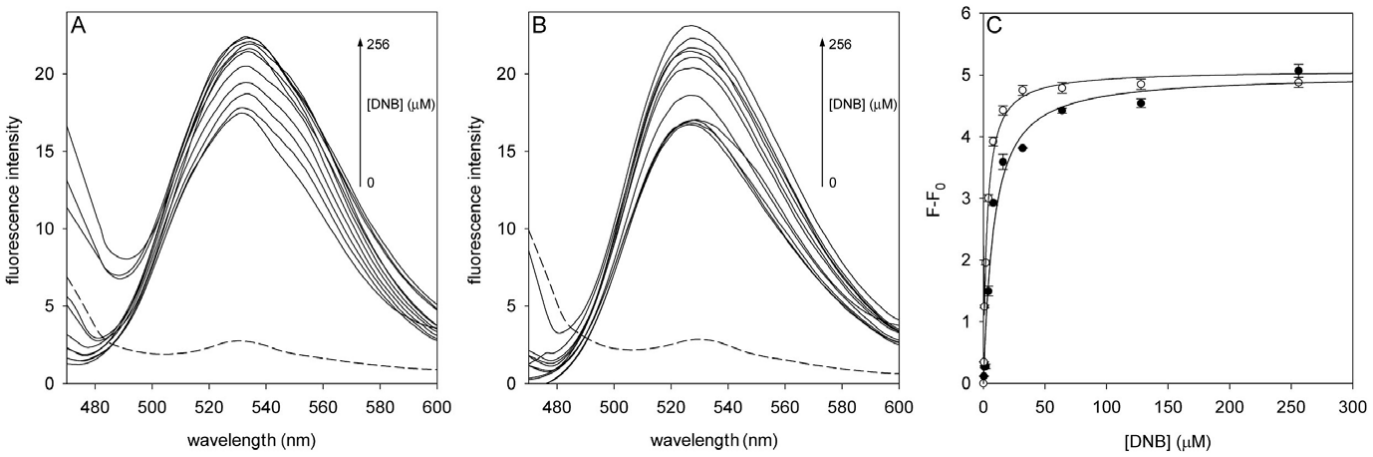
Titration of DprE1 with DNBs. Fluorescence spectra of DprE1 at increasing concentrations of DNB1 (A) and DNB3 (B); the fluorescence intensities are reported in arbitrary units. The dashed lines represent the fluorescence spectra of a blank sample containing 256 µM of ligand. All measurements were performed at 25°C, as reported in “Materials and Methods” section. (C) Titration curves of DprE1 with DNB1 (•), and with DNB3 (○). Lines are the fit of the data to equation 1.

DprE1 has been reported to have a FAD binding domain [10], and effectively the recombinant protein showed the typical fluorescence emission spectra of the flavin adenine dinucleotide (maximal emission at 530 nm with λ_ex_ = 450 nm) (Figure 1). To obtain direct evidence on the binding of DNBs to DprE1 the interactions between the two counterparts were investigated monitoring the change in fluorescence of the enzyme bound FAD, in the presence of different ligand concentrations. DNB1 and DNB3 were used for titrations, and in both cases the intensity of the fluorescence was enhanced by the ligand (Figure 1, A and B), showing a saturation curve (Figure 1C). Moreover, no alterations in fluorescence was found when dimethyl sulfoxide, the solvent of DNBs (see Materials and Methods), was used alone. The titration curves obtained were fitted to equation 1 (Figure 1C), and the Kd values determined (7.3±0.8 µM and 1.8±0.1 µM for DNB1 and DNB3, respectively) showed that DprE1 binds both DNB compounds with high affinity.

This result shows unambiguously that DprE1 is a target of DNB compounds.

### NfnB is responsible for DNB transformation

To verify whether DNB3 resistance in *M. smegmatis* DNB^R-Low^1-3 mutants was really associated to the overexpression of *nfnB*, quantitative Real Time PCR (qRT-PCR) experiments were performed on wild-type *M. smegmatis* and mutant strains in the absence of the drug. A statistically significant increase in transcription of *nfnB* was observed in DNB^R-Low^1-3 mutants with respect to the wild-type strain (Table 4). Moreover, the levels of *nfnB* overexpression detected in DNB^R-Low^1-3 mutants were higher than those of GM22, AL49 and AL55 mutants [14].

**Table 4:** *nfnB* expression levels obtained by Real Time PCR in different *M. smegmatis* strains.

*M. smegmatis* strain	*nfnB*
**mc^2^155**	1
**DNB^R-Low^1**	3907±307
**DNB^R-Low^2**	2755±271
**DNB^R-Low^3**	1602±209

Therefore, as in the case of BTZ low resistant mutants, the overexpression of the nitroreductase NfnB turns out to be involved in the mechanism of resistance of DNB^R-Low^ mutants.

To assess if the overexpressed NfnB was responsible for DNB inactivation, the purified enzyme was evaluated for its ability to convert the *nitro-*form of DNB1 into the corresponding *hydroxyl amine*- and/or *amino*-derivatives. The experiments carried out by LC/MS^2^ analysis were performed both in the presence and in the absence of NADPH, as described in Materials and Methods.

**Figure 2:**
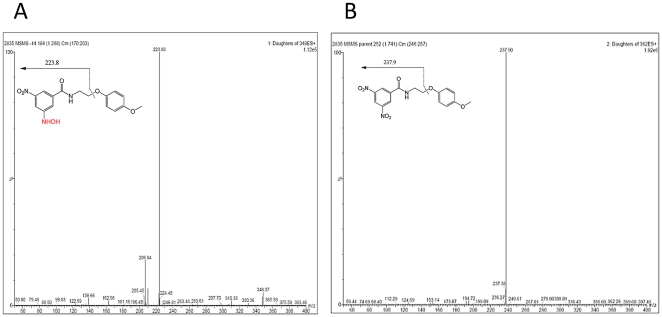
LC/MS^2^ analysis of DNB transformed by NfnB. DNB1 was incubated with *M. smegmatis* NfnB in presence of NADH and the mixture was analyzed by LC/MS^2^ A. DNB1; B. *hydroxyl-amine* form of DNB1 that is detected after a 30 min incubation at 37°C.

Whereas NfnB was able to convert DNB1 into the corresponding *hydroxyl amine*-derivative in the presence of NADPH (Figure 2A), no conversion was observed in the absence of this cofactor (Figure 2B). On the other hand, no formation of the *hydroxyl amine*-form was observed when NfnB was excluded from the reaction mixture, thus confirming the role of the enzyme in the drug transformation (data not shown). However, the DNB *amino*-derivatives were not detected in our experimental settings. On the other hand, previous studies performed on *M. smegmatis* extracts incubated with DNBs evidenced the production of both *amino*- and *hydroxyl amine*-derivative forms of the drug (data not shown), as in the case of BTZ compounds [8], [14]. Moreover, the *in vitro* synthesis of the DNB *amino*-derivative and *hydroxyl amine*-form were successful [15] and these forms of DNB were not effective against mycobacteria similarly to BTZ derivatives [8], [14], [15].

In conclusion, the data reported here elucidate the mechanisms of resistance of DNBs and BTZs in *M. smegmatis*. Accordingly, we have demonstrated that DNBs and BTZs have the same target and the same mechanisms of resistance, hinging on either the mutation of the target or the transformation of the original *nitro* compound into a less active *amino* or *hydroxyl amine* derivative.

In this work we only used *M. smegmatis* for our experiments for the following reasons:

1. The mechanism of resistance related to NfnB (drug inactivation) is present only in *M. smegmatis*. Until now in *M. tuberculosis* a nitroreductase able to inactivate the BTZ has not been found [14]. Moreover, the overexpression of *nfnB* in *M. tuberculosis* confers resistance to BTZ [14].
2. In our laboratory we tried to obtain soluble *M. tuberculosis* DprE1 soluble protein without success, but we obtained small amounts of *M. smegmatis* soluble protein that we used for the experiment of binding between DprE1 and DNB.
3. *M. smegmatis*, *M. tuberculosis*, and *M. bovis* BCG mutants resistant to BTZ have the same mutation in the same codon of DprE1.

The cellular target of DNB drugs in *M. smegmatis* is DprE1, wich is a component of the decaprenylphosphoryl-β-D-ribose 2′-epimerase, an enzyme involved in arabinogalactan and lipoarabinomannan synthesis in the mycobacterial cell wall. To accomplish their activity these compounds require the presence of a cysteine at position 394 of the *M. smegmatis* DprE1 enzyme. Substitution of this cysteine by glycine or serine in DprE1 affects the power binding of DNBs, thus making mycobacteria highly resistant to these drugs (DNB^R-High^). Moreover, direct evidence of interaction between DprE1 and DNBs was demonstrated by steady state fluorescence titration.

However, the resistance mechanisms can be developed indirectly, making the DNB molecules less effective through the reduction of their *nitro*-forms to *hydroxyl amine*- and/or *amino-*derivative. The resulting resistance is achieved through the overexpression of the gene encoding the nitroreductase NfnB, as a consequence of its mutated repressor. This last point was also demonstrated by LC/MS^2^ where NfnB transformed the *nitro* form of DNB into the *hydroxyl amine* inactive derivative.

Both groups of mutants (DNB^R-High^ and DNB^R-Low^) display a behavior towards DNBs similar to that exhibited to BTZs, indicating that the mechanism of action of DNBs parallels that suggested for BTZs.

Furthermore, this study presents some novelties: 1. DprE1 is a target of DNB compounds in *M. smegmatis* and, consequently, in all mycobacteria; 2. DprE1 binds the DNBs with high affinity; 3. in *M. smegmatis* NfnB is responsible also for DNB inactivation.The knowledge of the mechanisms of action and of resistance to BTZ and to DNB will help the design of derivatives less susceptible to nitroreduction and with a higher *in vivo* efficacy.
